# Do Doctors Have a Responsibility to Challenge the Distorting Influence of Commerce on Healthcare Delivery? The Case of Assisted Reproductive Technology

**DOI:** 10.1007/s10728-024-00500-3

**Published:** 2024-11-19

**Authors:** Craig Stanbury, Ian Kerridge, Ainsley J. Newson, Narcyz Ghinea, Wendy Lipworth

**Affiliations:** 1 Monash Bioethics Centre, Department of Philosophy, Monash University, Clayton, VIC 3800 Australia; 2 Faculty of Medicine and Health, Sydney School of Public Health, Sydney Health Ethics, The University of Sydney, Sydney, NSW 2006 Australia; 3 Macquarie University Research Centre for Agency, Values and Ethics, Department of Philosophy, Macquarie University, Macquarie Park, NSW 2109 Australia

**Keywords:** Commercial influences, Assisted reproductive technologies, Complicity, Moral responsibility, Collective action

## Abstract

Medicine has always existed in a marketplace, and there have been extensive discussions about the ethical implications of commerce in health care. For the most part, this discussion has focused on health professionals’ interactions with pharmaceutical and other health technology industries, with less attention given to other types of commercial influences, such as corporatized health services and fee-for-service practice. This is a significant lacuna because in many jurisdictions, some or all of healthcare is delivered in the private sector. Using the exemplar of Assisted Reproductive Technologies (ART), this paper asks: what, if any, responsibilities do doctors have to challenge the distorting influence of commerce in healthcare, other than those arising from their own interactions with health technology companies? ART provides a good focus for this question because it is an area of practice that has historically been provided in the private sector. First, we describe a range of concepts that offer helpful heuristics for capturing how and when doctors can reasonably be said to have responsibilities to resist commercial distortion, including: complicity, acquiescence, wilful ignorance, non-wilful ignorance, and duplicity. Second, we present ways that individual doctors can act to stop questionable behaviour on the part of their colleagues, clinics/corporations, and their profession. Third, we note that there are many situations where change cannot be achieved by individuals acting alone, and so we consider the responsibilities of health professionals as collectives as well as the role that professional bodies and regulators should play.

## Introduction

Medicine exists in a marketplace. For this reason, there have been extensive discussions surrounding the ethical implications of health professionals’ commercial practices, including their interactions with industry and the ways in which they are paid [1–5]. Much has been written about the responsibilities health professionals should uphold when it comes to managing their interactions with industry—for example, whether and how they should disclose interactions with industry that may cause, or be perceived to cause a conflict of interest [6, 7]. In contrast, far less attention has been given to health professionals’ responsibilities in regard to other types of commercial influences, such as their work within corporatized health services and fee-for-service practice. This is a significant gap because in many jurisdictions, some or all of healthcare is delivered in the private sector.

It is broadly agreed that doctors need to recognise that commercial forces can influence their decision-making. Doctors are expected to acknowledge their biases, be open and transparent, and focus on the best interests of their patients. They may also be expected to avoid certain types of employment or contractual arrangements that can compromise their independent clinical judgement. There is, however, far less agreement as to whether doctors have any obligations to speak out or act when they see commercially motivated actions on the part of their colleagues, the clinics for which they work, or their broader professional groups that have the potential to undermine clinical quality and safety, or otherwise impact negatively on the broader health system. This paper uses the example of Assisted Reproductive Technologies (ART) to critically consider the question: what, if any, moral responsibilities do doctors have to challenge the distorting influence of commerce in healthcare beyond those arising from interactions with industry?

ART provides a good focus for this question because it is an area of practice that has historically, in many countries, been provided in the private sector, even in jurisdictions in which some funding is provided by public payers [8]. In Australia, for instance, ART is a financially booming industry. Two major clinics, owned by private equity companies, were floated on the stock exchange in 2014 raising more than $300 million each [6], and the revenue in Australia from the ART industry was projected to reach $800 million in 2024 [9]. This private equity investment and attraction of venture capital has been welcomed by the financial sector, with one analyst commenting that ‘people will pay almost anything to have a baby’ [10].

While ART in Australia is widely regarded as being of high quality, concerns have been raised by clinicians, ethicists, and other individuals (such as patients or payers), about the effects of commercialisation on the quality and cost of service provision in ART [4, 5, 11–13]. There are, for example, concerns that corporatisation of ART has led, in some cases, to increased costs for patients, lack of personalisation of care [3, 8], and ‘over-servicing’, particularly with regard to premature commencement of in-vitro fertilisation (IVF) and continuation of IVF cycles when success is unlikely [11].

Specific concerns have also been raised about the use of particular interventions called ‘add-ons’ [14–18]. Add-ons are aimed at improving patient experience or birth outcomes but, by definition, they do not have sufficient evidence to be considered part of standard medical practice [14]. While they may be effective in defined subsets of patients, they can lead to increased psychological and biomedical harms with equivocal or little clinical value [14]. They also come with significant additional costs on top of standard IVF cycles - thus, having the capacity to cause financial harm [14, 19].

While add-ons can be viewed as a type of clinical innovation [14], distorting commercial influences also play a role in driving their production, marketing, and use [20–23]. Patients commonly learn of add-ons through a clinic’s marketing, then may demand the use of them and pay the extra costs despite a lack of evidence for their success [14]. When add-ons are used in this way—that is, driven by patient demand rather than clinical judgement—they may be unnecessary and costly interventions.

Given these concerns about the commercialisation of ART, ART provides an ideal case study to critically consider what moral responsibilities doctors may have to challenge the distorting influence of commerce in healthcare beyond those arising from interactions with industry. Importantly, by using ART as a case study, we intend for our analysis to be applied more generally to other areas of practice. The ideas we explore are not unique to ART and so our insights can be applied to areas of practice such as cosmetic surgery, stem cells, and complementary medicine.

In Section Defining the Parameters of Doctors’ Moral Responsibilities, we clarify the type of commercial influence that we are concerned with in this paper and analyse the parameters of a doctor’s moral responsibility to challenge distorting commercial influences. We argue that there are a range of concepts that can usefully assist in articulating doctors’ responsibilities, including complicity, acquiescence, wilful ignorance, and non-wilful ignorance. We show how complicity in particular offers a helpful heuristic for capturing how and when doctors can be reasonably said to have responsibilities to push back against commercial distortion in settings like ART provision. We then discuss the limitations of complicity and the need to draw on other concepts.

In Section How Doctors Can Fulfil Their Responsibilities to Act we examine what doctors can do to exercise their moral responsibilities. We present ways that individual doctors can speak up/act to stop questionable behaviour on the part of their colleagues, clinics/corporations, and their profession. We also note that there are many situations in which individuals can achieve very little, whereas collectives may have power to influence policy, practice and the actions of more powerful bodies (e.g., governments, companies or professional groups). We therefore also consider the responsibilities of health professionals as collectives and of professional bodies and regulators.

## Defining the Parameters of Doctors’ Moral Responsibilities

This paper addresses areas of practice where the moral status of commercial influence is unclear or contested.^1^ Our concern is not, therefore, with circumstances in which commercial influences are obviously morally justifiable—for example, where an ART specialist is using clinic resources to conduct high quality research that is approved by an institutional ethics committee and conducted at arms-length from the clinic. Our concern is also not with circumstances in which commercial influences are obviously morally wrong, such as commercially-motivated negligence and fraud. It is uncontroversial that these cases are ethically, professionally and often legally problematic, and that people who witness them should speak out and/or challenge them. If, for example, an ART specialist is knowingly and deliberately providing misleading information about success rates in order to attract patients, or forging consent forms in order to make embryos available for research, then those who witness such actions must speak out. The law and professional codes of conduct already offer guidance on how to respond to such cases, so they are not the focus of this paper.

In between these broadly construed ‘obviously good’ and ‘obviously bad’ cases are moral ‘grey areas’, where there is reasonable disagreement about whether commerce has a negative distorting effect on health. In the ART setting, there may be reasonable disagreement about whether commerce is distorting health care when it comes to the use of interventions—including both ‘standard’ procedures and ‘add-ons’—that are of unproven benefit (i.e. research has not established whether a specific add-on works in particular circumstances) but are requested or desired by informed patients and unlikely to cause significant harm. These types of circumstances are not the focus of this paper because more discussion needs to be had about the clinical or scientific ‘rights and wrongs’ of the situation before any discussion about moral responsibility for pushing back on them can begin (insofar as there is a problem).

Another type of moral ‘grey area’ is where general clinical and ethical norms would suggest that a practice is clinically inappropriate and morally problematic, but not to the extent that it meets the criteria for negligence or misconduct. Some uses of ‘add-ons’ would fit into this category—for example, where they are not clearly unsafe, but have been proven (either through research or through years of clinical use) to be ineffective and may cause inconvenience or financial harm to patients, and this does not seem to be being adequately pre-empted or addressed by those recommending them. Here, it is not clear what responsibilities other ART specialists (and medical professionals more generally) have to call out the practices they observe or hear about. It is this type of circumstance that raises important questions about the moral responsibility of clinicians to push back against distorting effects of commerce.

There are several potential ways to approach the question of what, if any, moral responsibilities doctors have to challenge the distorting influence of commerce. One fruitful approach is to explore notions of complicity and related concepts such as acquiescence and ignorance. Several scholars have offered helpful accounts of complicity. Iris Marion Young, for instance, offers a useful framework for looking at how individuals should take action to address structural injustices. On her account, even though one did not bring about the broad and problematic situation, there may be an obligation to fix it [24]. The complicity model we use below from Chiara Lepora and Robert E. Goodin [25] is particularly helpful because they, unlike Young (for instance), directly apply complicity to clinicians as a case study. Their model, therefore, is in many ways ‘ready’ or ‘purpose built’ to help with the situation that we are concerned with.^2^

### Complicity

According to Lepora and Goodin, complicity consists of knowingly being involved in a morally problematic situation without being the primary cause of that situation. Reaching the minimum threshold of complicity does not require sharing the intentions of the individual or group who has created the circumstance in the first place. Instead, simply knowingly and willingly contributing—regardless of purpose—is enough to satisfy the *actus reus* and *mens rea* conditions for complicity.^3^ Therefore, someone is complicit if their contribution to a situation is:

1. Neither involuntary nor accidental;
2. Made with knowledge or culpable ignorance of the *contributory role* of their actions;
3. Made with knowledge or culpable ignorance of the *broader problems* they are contributing to.

Lepora and Goodin offer a taxonomy for assessing the degrees of complicity according to three factors: responsibility, contribution, and shared purpose:

**Table:** 

Responsibility factor (RF)	Contribution factor (CF)	Shared purpose (SP)
Voluntariness Knowledge of contribution Knowledge of wrongness of principal wrongdoing	Centrality of contribution Proximity of contribution Reversibility of contribution Temporality Planning role Responsiveness of contributors to principals	Shared Purpose (Extent of overlap) Strength of shared purpose Action guidingness of shared purpose

Using this taxonomy as a guide, addressing the degree of complicity can be determined by asking a series of questions. First, regarding the ‘Responsibility Factor’, does the person know (or are they culpably ignorant) that they could be contributing to a broader problem, even if they had no hand in bringing that situation about? And, how voluntary is their contribution? Second, regarding the ‘Contribution Factor’, how significant is their contribution? Even if they are not in control of the situation, is their contribution giving access to, facilitating, or making possible the problematic situation? Third, regarding the ‘Shared Purpose Factor’, while sharing the purpose of the broader problem is not required for complicity, if someone does share the purpose, their degree of complicity increases. Therefore, to what extent do they share the purpose?

Knowingly and willingly contributing to a problematic situation—being complicit—is always *pro tanto* blameworthy. Being *pro tanto* blameworthy as opposed to overall (or all-things-considered) blameworthy is an important distinction to make. If someone is only *pro tanto* blameworthy, then it means that they are only blameworthy to a certain extent, or ‘as far as it can go’. It does not imply that when every consideration is taken into account (i.e., when all things are considered) that they are blameworthy. Indeed, nothing about being *pro tanto* blameworthy can indicate whether there are other morally relevant considerations at play that impact whether someone is *overall* blameworthy. Instead, it only indicates that there is a responsibility to act and show why being complicit—bearing some causal responsibility for the situation—is not ultimately overall blameworthy.

Another important distinction to make is that between responsibility in the sense of *causal responsibility* (i.e., blameworthiness) and responsibility in the sense of *responsibility to act.* Importantly, when someone has a *responsibility to act*, this does not mean particularly ‘strong’, or supererogatory actions are obligatory. Instead, having a responsibility to act simply means that a person has a responsibility to provide a *moral response* of some kind. A moral response could include, for example, challenging the situation, minimising the role one plays in causing or perpetuating the situation, or even simply declaring and justifying the situation and one’s role in it while trying to raise concerns as best one can. Either way, the key point is that causal responsibility requires a moral response that should be meaningful and appropriate and not tokenistic.

The way in which one responds to a situation in which one is complicit can, in turn, determine whether one is merely *pro tanto* blameworthy or whether one is all-things-considered blameworthy. Being complicit with a situation matters morally, and so it will always be *pro tanto* blameworthy, but whether it is all-things-considered blameworthy depends on the moral response given.

Applying this thinking to doctors working in commercial environments, the circumstances in which doctors can be complicit include not only ‘obviously bad’ cases (where complicity may in fact be too weak a concept to capture the moral problem) but also some ‘grey area’ cases. According to Lepora and Goodin’s model, doctors in a ‘grey area’ of commercial influence arguably fall between two thresholds. They are below the threshold for being primarily responsible or to blame for the distorting effects of commerce. Yet, they are above the threshold for not making any contribution to the situation. In other words, while the situation is not their fault, they contribute to its problems and thus find themselves in a position in which they need to provide a moral response. We can illustrate this in the ART context through the following case study:

A doctor works for an ART clinic that offers procedures for which there is limited or no evidence of effectiveness from high quality studies. While, as noted earlier, there is reasonable disagreement about how individual doctors and patients should make decisions about add-ons, there is broader agreement that such interventions should not be advertised in potentially misleading ways, or integrated into clinic policies in a manner that makes it difficult for individual doctors and patients to avoid using them. This doctor’s clinic advertises add-ons on its website, and its Board actively encourages doctors to use them routinely.

The first question that needs asking in relation to this case is: does the use of add-ons create a problem with which one might be complicit? As noted earlier, add-ons may be expensive and cause physical and psychological harm. Thus, while there might be a good reason for an individual doctor to offer a specific add-on to a particular (and properly informed) patient, a clinic that advertises add-ons for *all* patients, and/or actively encourages doctors to routinely offer them as part of clinic policy, represents a situation in which commerce is arguably having a distorting effect and in which clinicians may be complicit.

The second question that needs to be asked is: does the doctor know that the situation is morally problematic (i.e., ‘Responsibility Factor’)? It is reasonable to assume that doctors offering add-ons know when their clinics are advertising these interventions and when their Boards are actively encouraging their routine use. Even if the doctor does not participate directly in clinic marketing decisions or sit on the Board, they would know what is happening. And even if they do not necessarily share the commercial intentions of the clinic or board, knowingly and voluntarily being part of the organisation in which add-ons are advertised and their routine use actively encouraged, means that the doctor has the required *mens rea* to be complicit in their use.

Given that complicity involves a secondary contribution to something that one did not primarily bring about, the third question that needs to be asked is: how much of a contribution does the individual doctor make to the problematic situation (i.e., ‘Contribution Factor’)? It seems in this instance that the contribution is significant. While some ‘add-ons’ such as complementary medicines are widely available to patients, most cannot be offered without a doctor. The system, in other words, *relies* on doctors to realise its intentions. Therefore, a doctor offering unproven interventions in this context satisfies the *actus reus* required for complicity. They are culpably complicit with the commercial distortion in these ‘grey areas’ of ART.

Concluding that, in this case, the doctor is complicit with the distorting influence of commerce in ART means that they are *pro tanto* blameworthy and so have a moral case to answer (i.e., *a responsibility to act*).

### Beyond Complicity: Acquiescence, Ignorance, Duplicity

While cases such as the above describe situations in which doctors are complicit, there are other situations in which doctors bear some moral responsibility to act even if they do not meet the criteria for complicity. In these cases, it is necessary to draw on alternative concepts such as acquiescence (where a person knows about a problematic action and allows it without contributing to it causally), wilful ignorance (where a person does not know about a problematic action but arguably could or should know) and non-wilful ignorance (where information is withheld such that the person cannot be expected to know about it).

An example of acquiescence would be a situation in which an ART doctor works for a clinic that advertises questionable add-ons. The doctor does not use them in their practice, but knows that colleagues are using them and says and does nothing.^4^ A case of wilful ignorance would be a doctor who works for a clinic that advertises add-ons and who encourages their routine use, but does not know that these interventions are ineffective despite the fact that this is reasonable knowledge for a professional in their role to have.^5^ Of course, this type of case is potentially bordering on outright negligence. So it may be situated on the boundary between ‘grey areas’ and ‘obviously bad cases’ on the spectrum of commercial influence. Either way, the point is that there can be cases of wilful ignorance where a doctor does not have causal responsibility, yet still bears some moral responsibility to act. A case of non-wilful ignorance would be a doctor who works for a clinic that advertises an add-on and encourages their routine use, but does not know that these interventions are ineffective because a company is hiding negative information about them in internal documents or the grey literature.

Whenever a doctor is complicit, acquiescent or wilfully ignorant, they may also be duplicit, by which we mean that they actively conceal information. In the cases above, the doctor would be duplicitous if they exaggerated the efficacy of add-ons to patients or colleagues, or simply withheld information about them. Duplicity adds a layer of moral responsibility to any action.

## How Doctors Can Fulfil Their Responsibilities to Act

We have argued that doctors have a responsibility to act in situations where complicity, acquiescence, ignorance, or duplicity are at play. However, given that a doctor is only one individual in a complex and dynamic system, determining how they should act can be morally unclear. What exactly can an individual doctor do in such circumstances? Is our argument putting too much responsibility on individuals, effectively abandoning them to deal with what is arguably a complex structural issue?

One way to answer these questions, and so to understand how doctors can fulfil their moral responsibility to act, is to look at other instances where individuals can feel confused or lost when they find themselves part of, or complicit with, broader systemic problems. For example, there is extensive discussion on this point in environmental ethics [c.f. 28–32]. In the case of fighting against climate change, individuals can often feel powerless to do anything about the problem. Yet, there are still actions that they can take.

For instance, even though the actions of a single individual are insufficient to cause climate change, individuals can still attempt to minimise their contribution to it. People recycle for these very reasons. Individuals do not recycle because they believe that *their* recycling will save the planet, but because they believe it is good not to contribute to harming the environment [31]. At a collective level, if no one recycled, consumption would be even more unsustainable. Travis Rieder makes the point that while it may be impossible to live in such a way that one makes no contribution to climate change, there are strong reasons (such as not being complicit) in favour of understanding and taking responsibility for minimising one’s contribution (whatever that may amount to) to a problematic situation where it is feasible and not too demanding to do so [33].

Attempting to achieve herd immunity via vaccines works in a similar way. If *I* do not get vaccinated, nothing will change regarding the spread of a virus. Nevertheless, that is the wrong way to conceptualise my place in the complex web of social responsibilities. If everyone acted as I did, we would not achieve herd immunity. Therefore, even though I cannot achieve herd immunity on my own, I ought to, at the very least, take control of what I *can* do, for whatever that is and however far as that goes.

In any case, taking control of what one can do often makes a difference. Individual ART doctors, for instance, can speak up to the company, go against clinic policy, acknowledge their blindspots to patients, or deliberate in advance the most ethical way of speaking to their patients about commercial influence and distortion.^6^ Moreover, if a doctor sits on the Board of a clinic, they can help to reduce the risk of speaking up by making it difficult for superiors to punish doctors for doing so. Indeed, if they are in a position of leadership within the clinic, they can help to design a moral organisation. They can implement organisational ethics programmes to encourage their clinics to reflect on their values and aims, and how these aims can be ethically expressed through their governance structure, policies, and practices [34].

Nevertheless, a doctor may reasonably reply here that they still feel powerless to do anything as an individual. What, then, can they do? We believe that they can still provide a meaningful moral response and so fulfil their responsibilities by *collectivising*. Consider again an example from the environmental ethics literature. While individuals may feel powerless to mitigate climate change, collectives *can* make a difference [29]. For instance, if minimising the use of single-use plastics is important for sustainable living, one person refraining to use single-use plastics is not going to make a difference. Whereas if everyone banded together and refused to use these plastics or campaigned for them to stop being produced, meaningful change could occur. Therefore, because collective action does create meaningful change, environmental ethicists argue that individuals plausibly have an obligation to form into groups/collectivise to create this change. As Elizabeth Cripps says: “what should each of us do about climate change?… Focus on what you can achieve working with others who are also motivated by climate justice. Not on what you can do narrowly as an individual.” [29].^7^

While collectivisation may be difficult within a single commercial clinic or company, professionals have other avenues for collectivising. Blakely et al. [6], for example, suggest that within the ART context professional bodies can effectively act by becoming leaders in creating a systematic and probing *discourse* in their field. They can, for instance, put together conferences that include clinicians, patients, consumer groups, regulators and bioethicists all presenting and taking part in a conversation about how to best respond to their complicity with distorting commercial influence.

Professional bodies could also put together a series of questions that creates an ongoing forum about how to systematically address the issue of complicity. These questions, and this discourse, could include the following:

- How might commercial influences get in the way of fulfilling our professional obligations as doctors?
- What are the strengths and weaknesses of existing regulatory structures, policies and practices for the management of commercial influences in IVF?
- What are the responsibilities of the professional bodies within this context?
- How can the professional bodies best help individual doctors to avoid, or respond to, being complicit with distorting influence?
- How can we, as a collective, and as individuals, remain autonomous and committed to our professional values within a commercial context?

As a result of this discourse, professional bodies and institutions could then support development and implementation of more robust guidelines about complicity and commercial influence. (This assumes, of course, that the professional bodies are not, themselves, unduly influenced by the commercial interests that they are seeking to critique.)

## Conclusion

This paper has developed an account of doctors’ moral responsibilities when they are working in a commercial health sector, using Assisted Reproductive Technologies (ART) as an exemplar. Complicity and the related concepts of acquiescence, ignorance, and duplicity all provide valuable and helpful insights into how a doctor may be morally responsible for some of the negative impacts of commerce in medicine. They show that doctors have a responsibility to act—that is, provide some kind of moral response—to their involvement with problematic situations. While doctors do not necessarily need to do anything supererogatory (for example, taking actions that might otherwise compromise their job, career, or livelihood), *some* sort of response is required. Importantly, this response may need to be collective in order for it to be effective and needs to consist of a range of ethical procedures and structural changes.

## Footnotes

1 These distinctions are intended to be a heuristic to define our broad territory of interest and not a formal categorisation of moral rightness, wrongness and responsibility.

2 It has also been used elsewhere in the literature for addressing what clinicians should do in morally complex (and collective action problem) scenarios. c.f [26].

3 This is notably less stringent than a lot of legal doctrine. As Lepora and Goodin say, the *mens rea* often required for complicity in the law consists of ‘‘not only awareness but also fully joint action and a meshing of purposes’’. Nevertheless, we are not making any legal claims here and are instead interested in moral responsibility.

4 This falls short of complicity because there is no *actus reus.*

5 See David Luban for more on wilful ignorance within professional roles [27].

6 Bazerman [34] discusses these actions in the context of business ethics. Here, we have applied his insights to the concern of this paper.

7 Other circumstances in which collective action is both necessary and powerful include trade union activities, social advocacy against racism and other forms of discrimination.
